# Pioneering genome editing in parthenogenetic stick insects: CRISPR/Cas9-mediated gene knockout in *Medauroidea extradentata*

**DOI:** 10.1038/s41598-025-85911-5

**Published:** 2025-01-20

**Authors:** Giulia Di Cristina, Elina Dirksen, Benjamin Altenhein, Ansgar Büschges, Sigrun I. Korsching

**Affiliations:** 1 Institute of Zoology, Faculty of Mathematics and Natural Sciences, University of Cologne, Cologne, Germany; 2 Institute of Genetics, Faculty of Mathematics and Natural Sciences, University of Cologne, Cologne, Germany

**Keywords:** Parthenogenesis, CRISPR/Cas9, *Medauroidea Extradentata*, Neurogenetics, Eye pigments, Entomology, Molecular biology

## Abstract

The parthenogenetic life cycle of the stick insect *Medauroidea extradentata* offers unique advantages for the generation of genome-edited strains, as an isogenic and stable mutant line can in principle be achieved already in the first generation (G0). However, genetic tools for the manipulation of their genes had not been developed until now. Here, we successfully implement CRISPR/Cas9 as a technique to modify the genome of the stick insect *M. extradentata*. As proof-of-concept we targeted two genes involved in the ommochrome pathway of eye pigmentation (*cinnabar* and *white*, second and first exon, respectively), to generate knockout (KO) mutants. Microinjections were performed within 24 h after oviposition, to focus on the mononuclear (and haploid) stage of development. The KOs generated resulted in distinct eye and cuticle colour phenotypes for *cinnabar* and *white*. Homozygous *cinnabar* mutants showed pale pigmentation of eyes and cuticle. They develop into adults capable of producing viable eggs. Homozygous *white* KO resulted in a completely unpigmented phenotype in developing embryos that were unable to hatch. In conclusion, we show that CRISPR/Cas9 can be successfully applied to the genome of *M. extradentata* by creating phenotypically different and viable insects. This powerful gene editing technique can now be employed to create stable genetically modified lines using a parthenogenetic non-model organism.

**Supplementary Information:**

The online version contains supplementary material available at 10.1038/s41598-025-85911-5.

## Introduction

To understand complex biological pathways, genetic methods have proven invaluable, in particular for elucidating neural circuits and their role in generating behaviour. Genetic approaches allow visualization as well as activation or inhibition of specific neuronal populations in a manner not achievable by electrophysiological techniques alone. Currently the method of choice for gene editing is clustered regularly interspaced short palindromic repeats/CRISPR-associated protein 9 (CRISPR/Cas9), due to its precision, easy design and implementation, as well as cost-effectiveness. CRISPR/Cas9 has already been employed in many species beyond mouse, fruit fly, and zebrafish (see^1^ for review in arthropods). However, some excellent and well-established model systems to study the control of motor behaviour, such as Phasmatodea (stick insects), have not been subjected to genetic editing so far.

**Fig. 1:**
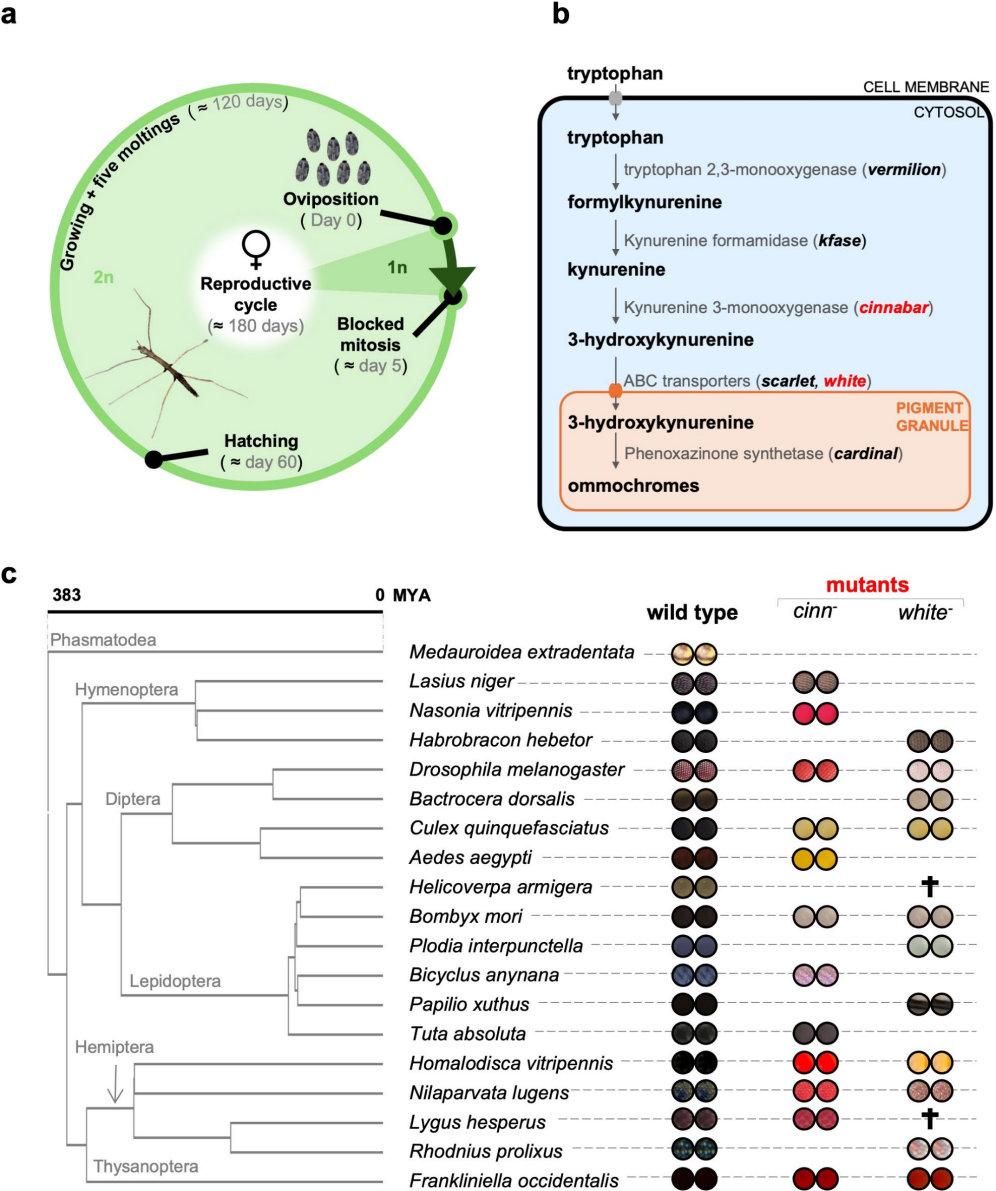
*Medauroidea extradentata* life cycle (**a**) and target genes selection (**b** and **c**). In (**a**) the reproductive cycle of a parthenogenetic female is outlined, oviposition to hatching, growing and reaching sexual maturity (about 180 days in total). The insect ommochrome eye pigmentation pathway (**b**) involves several genes (in bold) coding for different enzymes and transporters. Genes targeted in this study are red, and the eyes’ color resulting from their knockout in several insect species is reported in (**c**). The tree is built using the TToL5 web portal^48^; references for the colors corresponding to *cinnabar* and *white* mutations (*cinn*- and *white*-, respectively) are listed in Table S1. †: lethal recessive mutations.

Stick insects possess relatively small and accessible neural networks, which are highly suitable for the application of electrophysiological methods. The two species mainly used for these studies are *Carausius morosus* and *M. extradentata*. Both reproduce asexually by thelytokous parthenogenesis, i.e. mothers lay unfertilized eggs that are genetically identical to the mother. Of the two species, *M. extradentata* possesses a unique advantage for genome editing since it goes through a haploid phase until the 5th day after oviposition (Fig. 1a). From this point on, diploidy is restored by blocked mitoses: the chromosomes within the haploid nucleus duplicate but do not separate due to a spindle failure during metaphase, therefore they remain together and form a diploid nucleus^2^. Consequently, modification of the haploid nucleus before its first division will result in a homozygous mutation in the whole organism. Thus, an isogenic mutant line can already be established in the injected generation (G0). However, if desired, *M. extradentata* may also be induced to sexually reproduce allowing out and in-crossing^3^. As additional advantage, phasmids have a relatively low mutation rate^4^. Therefore, the traits introduced or modified through gene editing are likely to be more stable across generations.

Recently genomic and transcriptomic data of some phasmids including *M. extradentata* have become available^5–8^, simplifying the process of genome editing. We took advantage of this resource to implement CRISPR/Cas9 in *M. extradentata.* To easily identify the mutant stick insects, we targeted genes whose KO would produce a visible effect. These were *cinnabar* (*cn*) and *white* (*w*) (Fig. 1b), which have been intensively studied and targeted for KO in several insect species, producing different mutant eye colour phenotypes (Fig. 1c). *cinnabar* encodes the enzyme kynurenine 3-monooxygenase (KMO), that catalyses the reaction from kynurenine into 3-hydroxykynurenine, the precursor of different ommochromes^9^; *white* codes for an ABC (ATP synthase-binding cassette) transporter that functions in transporting pigment precursors into pigment granules^10^ (Fig. 1b).

**Fig. 2:**
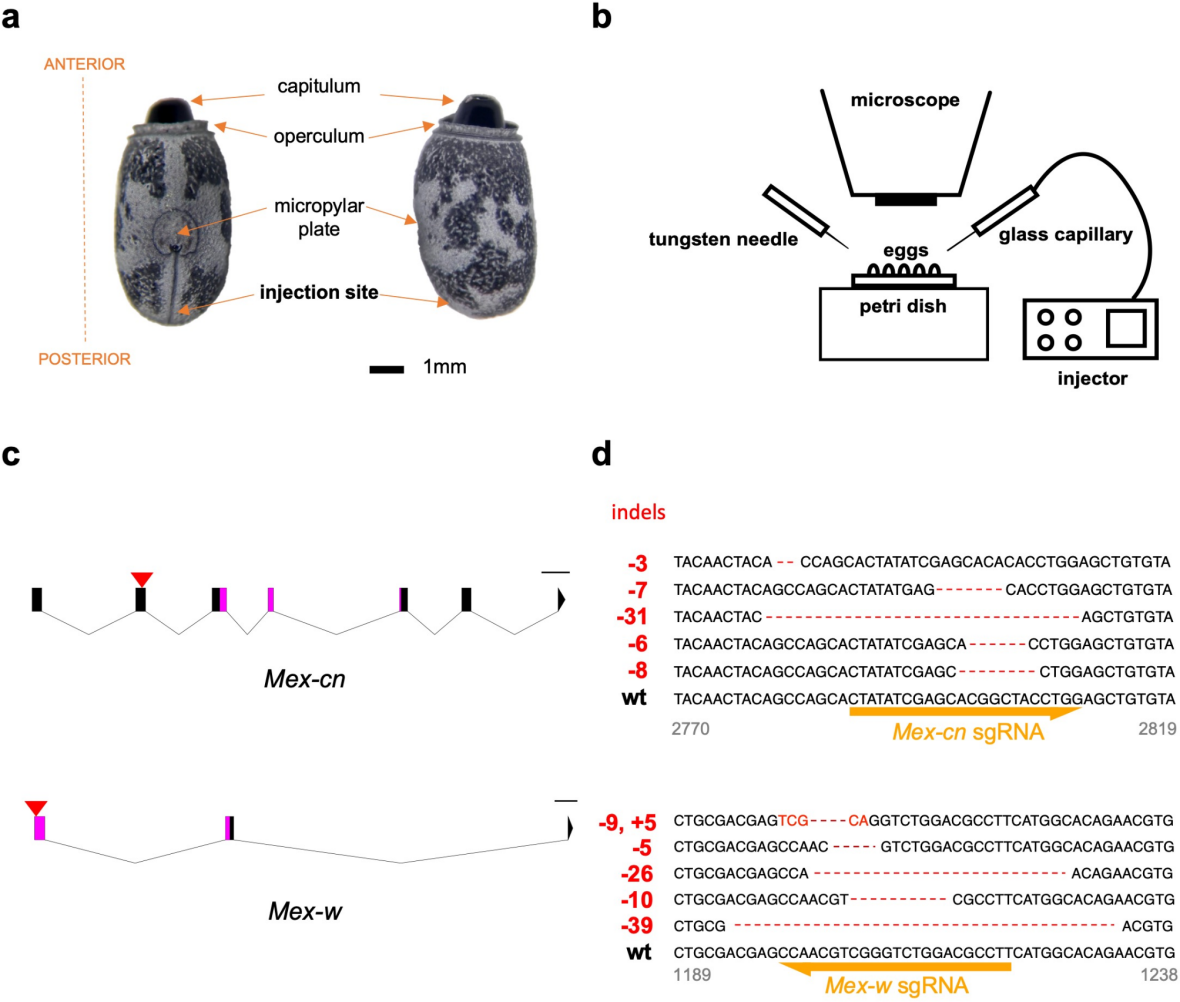
In (**a**) the egg of *M. extradentata* is shown from a dorsal (left) and lateral (right) view, in (**b**) a schematic representation of the injection set-up. The structures of *cinnabar* and *white* orthologs identified in *M. extradentata* genome are reported in (**c**), where the red arrow heads indicate the sgRNA target sites within exons and the predicted functional domain coding regions are in magenta (scale bar: 500 bp). (**d**) Target sequences from obtained mutant insects (G0) and wild-type controls are aligned; CRISPR/Cas9-mediated indels are shown in red. For details see text.

However, some specific characteristics of *M. extradentata* (and of stick insects in general) complicate the approach. The egg structure makes the delivery of the ribonucleoprotein (RNP) complex difficult. Although the large size of the eggs (Fig. 2a) eases their manipulation^11^, they have a relatively thick and hard exochorion, which makes the simple insertion of a glass capillary impossible. Additionally, the long period between oviposition and hatching (Fig. 1a) requires a method to prevent the yolk (and the embryo, successively) from completely drying out after the injection. Solving these problems allowed us to successfully generate homozygous knock-out insects, for the first time in any phasmid species.

## Results 

### Selected target genes *Mex-cn* and *Mex-w* show conserved genomic structures

 We identified *cinnabar* and *white* orthologs (*Mex-cn* and *Mex-w*) in the *M. extradentata* genome (see Methods). *Mex-cn* (PNEQ01077800.1 *Medauroidea extradentata* isolate BJ-2015 Med_ex_127824, whole genome shotgun sequence, 114743:124172) spans 9430 base pairs (bp) and contains 7 exons (Fig. 2c). The coding region (CDS) is 1167 bp long and encodes a protein of 389 amino acids (aa), with high similarity to KMO proteins in other insect species (47% with *Drosophila melanogaster*, 50% with *Aedes aegypti* and 47% with *Bombyx mori*). Exons 3–5 encode for a FAD binding domain (PF01494). *Mex-w* (PNEQ01053336.1 *Medauroidea extradentata* isolate BJ-2015 Med_ex_86787, whole genome shotgun sequence, 13706:25479) is 11,774 bp in length and has 3 exons (Fig. 2c). The CDS is 501 bp long and the encoded protein consists of 167 aa. The first two exons encode a P-loop NTPase fold (IPR027417) and are annotated as an ATP-binding cassette subfamily G transporter (IPR050352). Amino acid sequence comparison of White proteins between *M. extradentata* and other insects revealed a high level of sequence similarity (52% with *D. melanogaster*, 50% with *(A) aegypti* and 54% with *(B) mori*).

### Optimizing CRISPR/Cas9 injection parameters results in high mutation efficiencies for both *Mex-cn* and *Mex-w*

The viability results for treated and non-treated eggs are listed in Table 1. The eggs washed with 70% ethanol had a similar hatching rate and percentage of developed embryos compared to untreated eggs. Sham injections were performed with or without sealing the opening in the exochorion with glue after the procedure. Sham injections without glue sealing of the hole showed extremely low percentages of hatched eggs and developed embryos, however, this could be improved about tenfold by glue sealing the eggs after injection. No deleterious effect was observed by injecting the single guide RNAs (sgRNAs) for *Mex-cn* and *Mex-w* in comparison to the sham injection. However, the injection of Cas9 produced lower hatching and embryo development rates, in the range of 20–30% of the sham injection values.

**Table 1:** Viability of injected eggs and control groups. *First number gives the percentage of hatched eggs, number in parentheses gives the absolute number.

Treatment	sgRNA (ng/µl)	Cas9 (ng/µl)	Treated eggs	Hatched eggs % (*N*)*	Developed embryos** % (*N*)*
No treatment	n.a.	n.a.	1568	64.0 (1004)	70.1 (1099)
EtOH 70% washing	n.a.	n.a.	854	56.0 (478)	68.6 (586)
Sham injection	0	0	173	10.4 (18)	18.5 (32)
Sham injection w/o glue	0	0	160	0.6 (1)	2.5 (4)
only sgRNA - *Mex-cn*	400	0	216	7.4 (16)	25.0 (54)
only sgRNA - *Mex-w*	400	0	209	7.7 (16)	32.5 (68)
only Cas9	0	300	239	3.3 (8)	4.2 (10)
*Mex-cn* C1	500	300	235	2.1 (5)	8.9 (21)
*Mex-cn* C2	400	200	208	3.4 (7)	14.9 (31)
*Mex-cn* C3	400	300	490	2.2 (11)	21.0 (103)
*Mex-cn* C4	300	300	242	6.2 (15)	13.6 (33)
*Mex-w* C3	400	300	713	1.1 (8)	8.0 (57)

**Dissected 3–4 weeks after expected hatching time.

The combined injection of sgRNA and Cas9 resulted in roughly similar percentages of hatched eggs for both genes compared to the Cas9 injection, whereas the percentage of developed embryos was even higher for all but the highest sgRNA concentration. We evaluated several different concentrations for viability. The highest sgRNA concentration used (500 ng/µl) seemed to result in reduced viability, whereas the lowest concentration (300 ng/µl) resulted in maximal percentage of hatched eggs. Reduction of the Cas9 concentration from 300 to 200 ng/µl did improve the percentage of hatched eggs slightly. Next, we evaluated the efficiency of transgenesis for all combinations of sgRNA and Cas9 concentrations. The concentrations established as optimal for hatchlings viability (C4, 300 ng/µl sgRNA and 300 ng/µl Cas9) failed to produce any mutant hatchlings (Table 2).

**Table 2:** Mutation efficiency (number of gene edited samples/total number of samples), based on the phenotype and the genotype of embryos and 1st instar nymphs from the eggs injected with *mex-cn* and *Mex-w* sgRNA and Cas9.

	Phenotypes		Genotypes	
	Embryos	1st instars nymphs	Embryos	1st instars nymphs
*Mex-cn* C1	1/5*	0/5	15/16	0/5
*Mex-cn* C2	0/24	0/7	n.a.	n.a.
*Mex-cn* C3	43/92	3/11	n.a.	4/11
*Mex-cn* C4	5/11	0/15	n.a.	0/15
*Mex-w* C3	45/47*	0/8	25/26	0/5

C1: sgRNA 500 ng/µl and Cas9 300 ng/µl, *some embryos were too young for phenotype analysis; C2: sgRNA 400 ng/µl and Cas9 200 ng/µl; C3: sgRNA 400 ng/µl and Cas9 300 ng/µl; C4: sgRNA 300 ng/µl and Cas9 300 ng/µl.

**Fig. 3:**
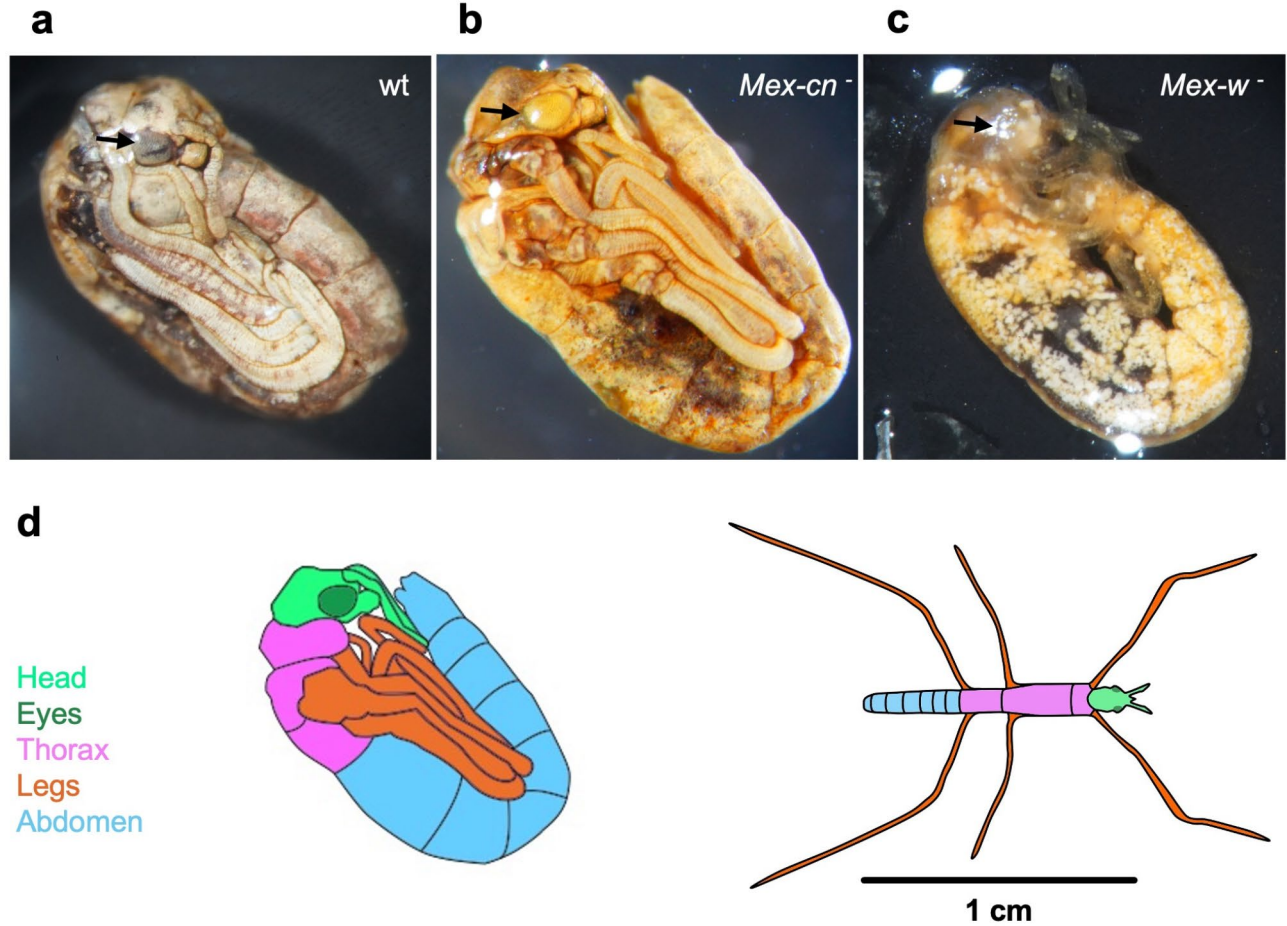
Pictures of embryos from wild-type and treated eggs, injected with Cas9 and either Mex-cn or Mex-w sgRNA dissected after the expected hatching timeframe (**a**–**c**); black arrows indicate the eyes. A legend to distinguish the different body parts is depicted in (**d**).

When injecting 500 ng/µl of *Mex-cn* sgRNA (C1), 94% of genotyped embryos showed an edited sequence, and when using 400 ng/µl of the same sgRNA (C3), 47% of dissected embryos had a clear phenotype (white eyes, Fig. 3). This percentage lowers to 46% when using the lowest *Mex-cn* sgRNA concentration (C4). Among the tested concentrations of Mex-cn sgRNA, C3 (400 ng/µl sgRNA and 300 ng/µl Cas9) offered the best balance between efficiency and viability. Therefore, we chose to use C3 as a starting point for injecting Mex-w sgRNA. This resulted in a lower hatching rate (1%) and embryo development rate (8%), compared to *Mex-cn* sgRNA used at the same concentration. The 96% of embryos dissected from eggs injected with Cas9 and *Mex-w* sgRNA showed a clear phenotype (transparent cuticle, see Fig. 3), and 25 out of 26 genotyped embryos (96%) were mutants. However, all 1st instar nymphs hatching from the eggs injected with *Mex-w* sgRNA were wild type (Table 2), suggesting a lethal effect of the mutation in late embryogenesis.

None of the potential off-target sites for *Mex-cn* and *Mex-w* were modified (for details, see Methods) suggesting that the observed phenotypes result from the mutations introduced in these two genes.

### CRISPR/Cas9-induced mutations result in visible phenotypes and are transmitted to the germline

**Fig. 4:**
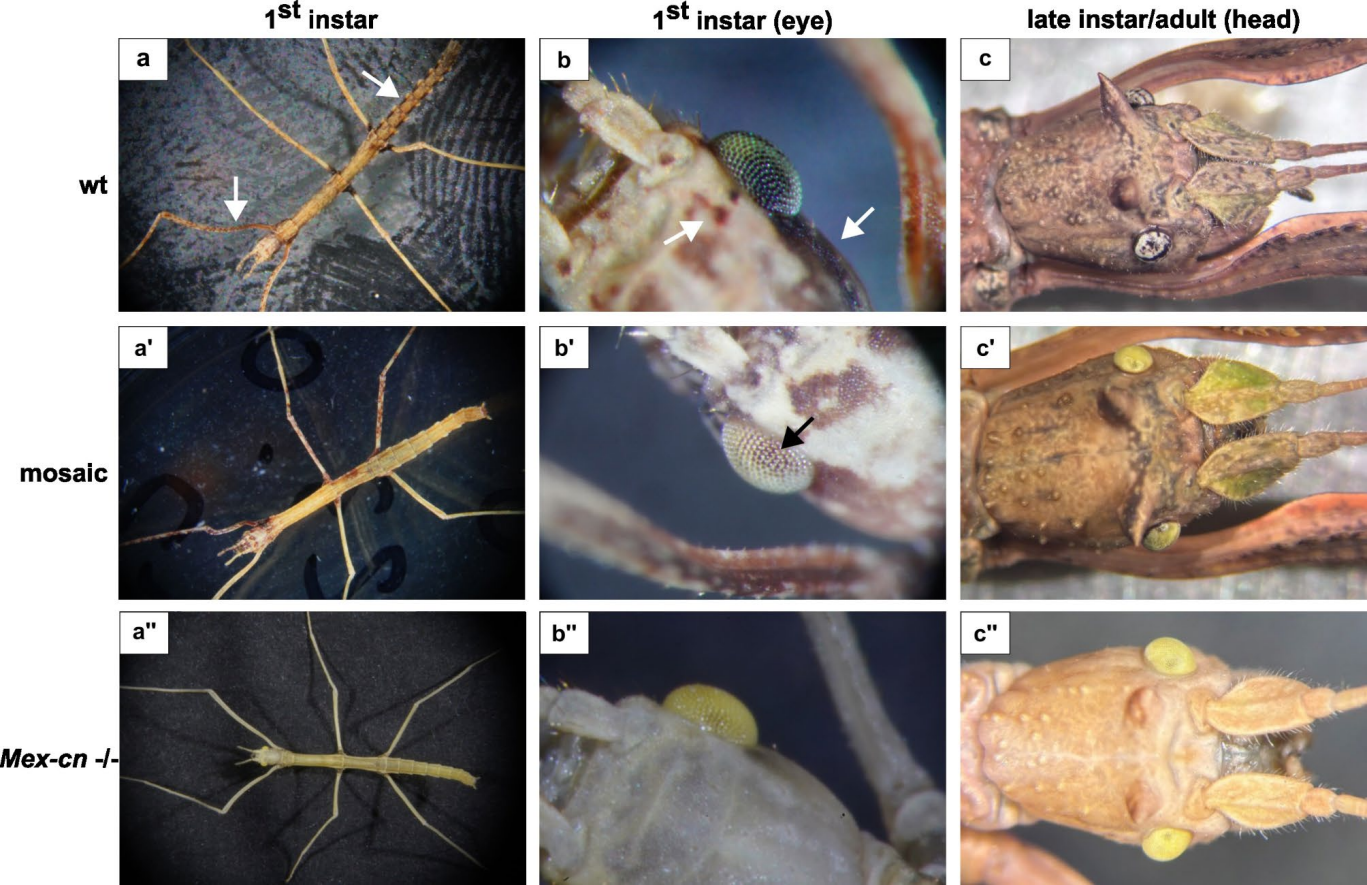
Phenotypes of wild-type and *Mex-cn* KO *M. extradentata* instars (**a**–**b**) and adult heads (**c**). White and black arrows point pigmented spots.

 G0 embryos dissected 90 days after injection already showed differences in their phenotype (Fig. 3a–c). Wild-type embryos (Fig. 3a) have brown pigmentation spots on the cuticle covering the head, thorax, legs and abdomen (Fig. 3d). Their eyes show a greenish background with darker spots with a horizontal line in the median part of the eye (Fig. 3a, arrow). Embryos with Cas9-induced indels in *Mex-cn* (Fig. 2d) sequence have pale cuticle and the eyes lack pigmentation. Most of them did not manage to hatch completely, but one succeeded, reached adulthood and reproduced. This insect showed a mosaic phenotype (Fig. 4a′–b′): it had overall lighter pigmentation, with a non-symmetrical bilateral distribution of pigments (Fig. 4a′). The eyes were mostly white, with a few brown spots on the dorsal part (Fig. 4b′). Its haemolymph was sampled and used for genotyping, and the result confirmed the presence of genetic mosaicism, with wild-type and two additional edited sequences of *Mex-cn* (4del, 63 del, Fig. S1). This insect successfully reached adulthood and exhibited brown cuticle pigmentation, which was slightly lighter than the wild type, accompanied by pale eyes. A small percentage (< 5%) of the over one thousand eggs laid by this individual hatched and produced viable offspring (G1). All offspring displayed a completely unpigmented cuticle and eye phenotype (Fig. 4a″–c″). Genotyping of 17 offspring revealed that they each carried a single edited *Mex-cn −/−* sequence, with overall five distinct CRISPR/Cas9-induced *indels* identified. (30in58del, 8in23del, 31del, 4del, 63 del, Fig. S1). Our mutant G1 population matured to adulthood, laid viable eggs, and produced a G2 generation that is phenotypically identical to G1.

Wild-type 1st instar nymphs show a pigmentation pattern similar to the embryo. They have dark spots on the lateral and dorsal surface of the head capsule (Fig. 4b, white arrows), dark stripes on the lateral sides of the abdomen and on the legs (Fig. 4a, white arrows).

Embryos with homozygous mutations in *Mex-w* (Fig. 2d) have completely transparent cuticle (Fig. 3c) and none of them reached the hatching stage. Similar lethality for *white* KOs has been reported in other species (see discussion).

## Discussion

Our results provide a proof-of-concept for the application of the CRISPR/Cas9 method in Phasmatodea, which are parthenogenetic non-model insects. As such, our results represent an essential milestone towards the application of molecular methods and gene editing in this species group.

Egg injections of the RNP complex led to a highly efficient mutation rate in *M. extradentata* embryos. To get the best compromise between efficiency and viability, different concentrations of sgRNAs and Cas9 were tested. As it has been shown before in *D. melanogaster*, increasing sgRNA concentrations go in hand with a higher editing efficiency, yet this can lead to decreasing viabilities^12^. According to our results, the optimal concentrations to balance viability and transformation efficiency is around 400 ng/µl for sgRNA and not less than 300 ng/µl for Cas9. This is similar to the optimal RNP concentration reported for *D. melanogaster*^12^. Future studies could explore the use of expression plasmids for sgRNAs and Cas9 as an alternative to RNPs. One study on *Tribolium castaneum*^13^ demonstrated that plasmids offered higher targeting efficiencies and improved survival compared to in vitro-synthesized sgRNAs.

We found it necessary to separate the opening of the eggshell from the actual injection, using a tungsten needle for the former and the usual glass capillary for the latter. Furthermore, it was essential to seal the injection hole immediately after the needle is removed, to avoid desiccation of the yolk during the long incubation period of the eggs. In fact, causing an opening in the eggshell produces a viability that drops dramatically from 10.4% in the sealed condition to 0.6% when the opening is not carefully closed with glue. Similar observations have been reported for *B. mori*^14^ and *Pyrrhocoris apterus*^15^, who also lay eggs in dry environments.

Both *Mex-cn* and *Mex-w* sgRNAs resulted in efficient editing of the respective target genes. Sequencing of the sgRNA target sites showed indel mutations of the genomic sequence that encode predicted non-functional proteins. These mutations are mostly homozygous, a fact that supports Bergerard’s reported haploidy of embryos in the first few days after oviposition^3^. In some cases, the mutations were detected only on one allele (the other one being wild type), or two different mutations were present on the two alleles. One possible explanation could be that the egg was provided with the RNP only after the first nuclear division, or the RNP complex was active throughout the process of the first division, also editing the daughter nuclei. Potential off-targets of *Mex-cn* and *Mex-w* sgRNA down to a predicted cutting probability of 2% were sequenced and none of them was modified, showing that both selected sgRNAs did not produce accidental off-target events in the predicted sequences.

The mutations are associated with a distinct phenotype for each gene. *Mex-cn* KOs showed a very pale pigmentation, suggesting that the enzyme encoded by *Mex-cn*, KMO, is probably not only involved in eye pigmentation, but also in the biosynthesis of cuticle pigments in *M. extradentata*. *cinnabar* knockout/knockdown is known to affect the eye and eggshell pigmentation in *B. mori*^16,17^ and influences eye colour in different insect species, causing red pigmentation in all hemipteran species studied so far^18–21^, white eyes in two dipteran species (*Aedes aegypti* and *Culex quinquefasciatus*)^22,23^ but not in *D. melanogaster* (where the eyes are red due to the presence of a second pigmentation pathway)^24^, and diverse eye pigmentation/mosaics in lepidopteran species^17,25–27^. *cinnabar* knockdown also produces effects on the cuticle of *Rhodnius prolixus*^28^. Thus, it is likely that KMO has slightly different roles in different insect orders, and that its KO leads to a broad range of effects, not only restricted to eye colour, but also including cuticle. In beetles, cuticle pigmentation is critical for optimal sclerotization (hardening of the exoskeleton)^29^. According to our results, KMO is responsible for the pigmentation of the eyes and the exoskeleton in *M. extradentata*. This might suggest that the kynurenine pathway confers some rigidity to the cuticle of *M. extradentata*, making it difficult for the *Mex-cn* KO 1^st^ instars nymphs to punch through the eggshell. This would account for the low hatching rate of *Mex-cn* KO insects. Sublethal fitness effects were observed when editing *cinnabar* in the mosquito *C. quinquefasciatus Say*^23,30^, thus contributing to a potential involvement of KMO in general fitness and ability to develop correctly. Loss of pigmentation has also been shown to be associated with reduced resistance to desiccation in *Aedes*, *Anopheles*, and *Culex*^31^. Lastly, we cannot rule out the impact of so far unrecognized off-targets, both because the genome is not in final stage, and because off-targets remain challenging to predict.

*Mex-w* KOs had transparent cuticle and were not viable. *white* KO produced complete loss of eye pigmentation in several insect species^20,32–37^, but also transparent/translucent cuticle in the milkweed bug *Oncopeltus fasciatus*^38^. Additionally, it has shown to be lethal in a few studies^18,38,39^ and has a clear effect on fitness in *Drosophila* too^40^. ABC transporters have several functions in arthropods^41^, and the one encoded by *white* is involved in a multitude of biological processes, indicating that its substrate specificity is not limited to pigment precursors. According to our results, *Mex-w* appears to be necessary for embryo survival.

The low hatching rate and viability of the Mex-cn -/- embryos is likely a consequence of the already mentioned fitness effects of cinnabar KO or conceivably related to so far unpredicted off-target activity of the sgRNAs, rather than the injection procedure. Nevertheless, we successfully generated a mosaic insect that produced a line of KO offspring. In fact, intentionally reducing the efficiency of gene editing to obtain mosaic insects might alleviate the problem of homozygous lethal mutations in future approaches.

Interestingly, the KO of a different gene (the myoinhibitory peptide gene; Dirksen et al., in prep.) showed a hatching rate reaching almost 20% of injected eggs, which is comparable or higher than many previous results obtained for other insect species^17,21,30,36,42^. Our optimized methods produced efficient and on-target gene editing that is inherited in all subsequent generations. This achievement serves as proof-of-concept for the use of CRISPR/Cas9 in stick insects and paves the way for applying genetic tools to study their biology. From now on, it will be possible to apply and further develop our optimized technique to generate mutant stick insects, both for knockout studies of the function of specific genes (e.g. their role in modulation of motor control) and for knock-in studies to label specific populations of neurons, or to selectively activate/inactivate them.

## Methods

### *Medauroidea extradentata* rearing

A parthenogenetic colony was maintained in cages inside a walk-in chamber set at 25 ± 1 °C, 50–60% humidity, with a 12:12 (L: D) h photoperiod. Insects were fed ab libitum with fresh wild blackberry leaves (*Rubus ulmifolius*).

### Identification and sequence analysis of *Mex-cn* and *Mex-w*

 The available genome assembly of *M. extradentata* (GenBank: GCA_003012365.1) was searched for putative orthologs of *cinnabar* and *white* genes (respectively, *Mex-cn* and *Mex-w*) using the BLASTn algorithm and respective mRNA sequences from other insect species as queries (see Table S1 for complete list and GenBank accession numbers). The resulting scaffolds with the lowest E-values were analysed with GenScan^43^ to predict coding sequences and translated peptides. The resulting peptides were blasted (BLASTp) against the NCBI database to identify those with high similarity to the protein encoded by *cinnabar* and *white* in other species. The first hit (E-value: 1e^− 174^) for the protein encoded by *Mex-cn* was kynurenine 3-monooxygenase in *Bacillus rossius* (XP_063243717.1). The first hit (E-value: 7e^− 77^) for the protein encoded by *Mex-w* was a protein white-like in *B. rossius* (XP_063236423.1). The identified protein sequences were analysed using InterProScan^44^ to identify conserved functional domains.

Total RNA was extracted and purified from adult tissues and first instars with the Monarch^®^ Total RNA Miniprep Kit (NEB T2010). 500 ng of extracted RNA were used as template for cDNA synthesis (ProtoScript^®^ First Strand cDNA Synthesis Kit, NEB E6300). Specific PCR primers (Table S2) were designed, and PCR amplification of *Mex-cn* and *Mex-w* was performed using Q5^®^ High-Fidelity DNA Polymerase (NEB M0491). The amplified fragments were purified using Monarch^®^ PCR & DNA Cleanup Kit (NEB T1030) and Sanger sequenced (Microsynth, Göttingen, Germany).

### Design and synthesis of sgRNAs

 sgRNAs targeting *Mex-cn* and *Mex-w* were designed and screened for potential off-target cleavage using the program CRISPOR^45^. To enhance the probability of efficient KO mutations, sgRNA target sites were designed to cleave within or upstream of regions coding for functional domains. Among the ten highest scoring sgRNA candidates we removed those that have off-targets above CFD (cutting frequency determination) of 0.02 in coding regions (as predicted by Genscan). The remaining candidates were synthesized and tested for in-vitro efficiency. DNA templates for sgRNA transcription were synthesized by Invitrogen (Thermo Fisher Scientific, Waltham, MA, USA). All DNA templates consisted of the 5′ T7 Polymerase promotor sequence (5′-TTCTAATACGACTCACTATA-3′), the 20 bp specific target site (Table S1) and a 14-nucleotide overlap sequence complementary to the *Streptococcus pyogenes* Cas9 scaffold oligo (GTTTTAGAGCTAGA). SgRNAs were synthesized with the EnGen^®^ sgRNA Synthesis Kit, *S. pyogenes* (NEB E3322S) and purified using the Monarch^®^ RNA Cleanup Kit (50 µg) (NEB T2040L). To test sgRNA in-vitro efficiency, suitable amplicons were amplified by PCR from genomic DNA (gDNA). The purified PCR products (120 ng) were then digested at 37 °C for 2 h in a mix containing 200 ng of the candidate sgRNA, 300 ng of EnGen^®^ Spy Cas9 NLS (NEB M0646M) and 1X NEBuffer r3.1 (NEB B6003S). The digestion products were separated and visualized in a 2% agarose TAE (Tris-acetate-EDTA) gel. The cleavage efficiencies were assessed comparing the amount of digested and un-digested products on the gel.

### Microinjection

The injection mixture consisted of the RNP complex of specific sgRNA and EnGen^®^ Spy Cas9 NLS. Cas9 and sgRNA were diluted in nuclease-free water at various concentrations (see Table 1) and preincubated for 15 min at RT for complex formation.

To achieve the highest chances of creating homozygous KOs resulting from injecting into embryos containing only one (haploid) nucleus shortly after oviposition, *M. extradentata* wild-type eggs were collected every morning (within 24 h after oviposition). We selected this timing based on a study of *C. morosus*, a stick insect species with a life cycle closely resembling that of *M. extradentata*, which showed that the first nuclear division in the egg happens after this period^46^. In preliminary experiments we found it necessary to briefly wash them in 70% ethanol to prevent contamination after injection. Eggs were placed in rows within BluTack (Bostik Limited, UK) and placed on an elevation under a microscope. Glass needles were prepared by pulling glass capillaries (WPI #504949) using a Next Generation Micropipette Puller (P-1000, Sutter Instrument, Novato, CA). Each needle was first backfilled with mineral oil (Sigma Aldrich, M5904-5ML). It was subsequently mounted on a microinjector (Nanoliter 2000/2020, WPI, Sarasota, FL) and approximately 3 µl of mineral oil were ejected. The needle’s tip was then inserted in a tube containing the injection solution, and the latter was then sucked into the needle at the speed of 200 nl/s. After the exochorion was carefully pierced at the posterior pole of the egg by a tungsten needle, 10–14 nl of injection solution were injected through the resulting hole (Fig. 2). The hole was sealed (Loctite AA 3321 LC) from the outside with light-cure adhesive, which was polymerized by approximately 1 s exposure to UV light. Eggs were kept inside petri dishes, with modified lids consisting of a fine-mesh net to allow airflow. Dishes were stored inside an incubator at 25 °C, with 60% humidity. The whole procedure is shown in snapshots in Fig. S2.

Control injection mixtures included sgRNA only or Cas9, both diluted in nuclease-free water. Sham injections were performed following the same procedure as the others, omitting the injection of solution. Untreated eggs and eggs briefly washed in 70% ethanol were additional control groups.

### Genomic DNA extraction and identification of Cas9-induced mutations

gDNA was extracted from randomly selected experimental samples (embryos, first instars, adult haemolymph). Whole embryos and first instars (single legs or whole body) were processed using the Monarch^®^ Genomic DNA Purification Kit (NEB T3010L). Haemolymph was sampled from the adult abdomen with a hypodermic syringe, and gDNA was purified using the DNeasy^®^ Blood & Tissue Kit (Qiagen 69504), following the protocol for nucleated erythrocytes. Briefly, 20 µl of Proteinase K were added to 5 µl of haemolymph and the volume was adjusted to 220 µl with Phosphate-buffered saline (PBS). Samples were then treated according to manufacturer’s instructions. *Mex-cn* and *Mex-w* DNA sequences were amplified from gDNA using Q5^®^ High-Fidelity DNA Polymerase and gene-specific primers amplifying a region around the expected cutting site. For primer sequences see Table S2. PCR products were visualized by gel electrophoresis, purified using the Monarch^®^ PCR & DNA Cleanup Kit (NEB #T1130) and Sanger-sequenced (Microsynth, Göttingen, Germany). Homozygous sequence results were analysed by aligning them to the wild-type genomic sequence using the Auto-MAFFT algorithm^47^ on Benchling (https://www.benchling.com/), while sequences showing more than one mutation/allele were manually annotated by analysing the chromatograms displayed on FinchTV 1.4.0 (Digital World Biology, Seattle, WA USA). Predicted potential off-target sites for each sgRNA were also amplified with specifically designed primers (Table S2) and analysed, to monitor potential unspecific induced mutations.

## Electronic supplementary material

Below is the link to the electronic supplementary material.

Supplementary Material 1

## Data Availability

Transcripts (mRNA) sequences for Mexcn and Mexw have been deposited in GenBank under the accession numbers PQ557935 and PQ557934, respectively. All other data and information are available from the corresponding author upon reasonable request. For inquiries, please contact gcristin@uni-koeln.de.
