# Publisher Correction: Pioneering genome editing in parthenogenetic stick insects: CRISPR/Cas9-mediated gene knockout in *Medauroidea extradentata*

**DOI:** 10.1038/s41598-025-93624-y

**Published:** 2025-03-20

**Authors:** Giulia Di Cristina, Elina Dirksen, Benjamin Altenhein, Ansgar Büschges, Sigrun I. Korsching

**Affiliations:** 1 Institute of Zoology, Faculty of Mathematics and Natural Sciences, University of Cologne, Cologne, Germany; 2 Institute of Genetics, Faculty of Mathematics and Natural Sciences, University of Cologne, Cologne, Germany

Correction to:* Scientific Reports* 10.1038/s41598-025-85911-5, published online 20 January 2025

In the original version of this Article Ansgar Büschges and Sigrun I. Korsching were omitted as equally contributing authors.

The original Article has been corrected.

