# Supplementary material for: Pioneering genome editing in parthenogenetic stick insects: CRISPR/Cas9-mediated gene knockout in Medauroidea extradentata
Source: Sci Rep. 2025 Jan 20;15:2584. doi: 10.1038/s41598-025-85911-5 (PMC11747256; doi:10.1038/s41598-025-85911-5)
Supplement: Supplementary file 1 — Supplementary Material 1 [file 41598_2025_85911_MOESM1_ESM.pdf]

SUPPLEMENTARY INFORMATION

Pioneering Genome Editing in Parthenogenetic Stick Insects: CRISPR/Cas9-Mediated gene knockout in *Medauroidea extradentata*

Giulia Di Cristina [1, *], Elina Dirksen [1], Benjamin Altenhein [1], Ansgar Büschges [1,3], Sigrun I. Korsching [2,3]

[1]Institute of Zoology, Faculty of Mathematics and Natural Sciences, University of Cologne, Cologne, Germany.

[2]Institute of Genetics, Faculty of Mathematics and Natural Sciences, University of Cologne, Cologne, Germany.

[3]Shared senior authorship: Ansgar Büschges and Sigrun I. Korsching.

* corresponding author: gcristin@uni-koeln.de

Table of contents:

Figure S1. Genotype of F1 generation *Mex-cn* mutants

Table S1. Accession numbers and references for Fig. 1c of main manuscript

Figure S2. Snapshots of injection procedure

Table S2. List of primers used in this study

SI text. Coding sequence for *Mex-cn* and *Mex-w*

Figure S1. Genotype of F1 generation *Mexcn* mutants

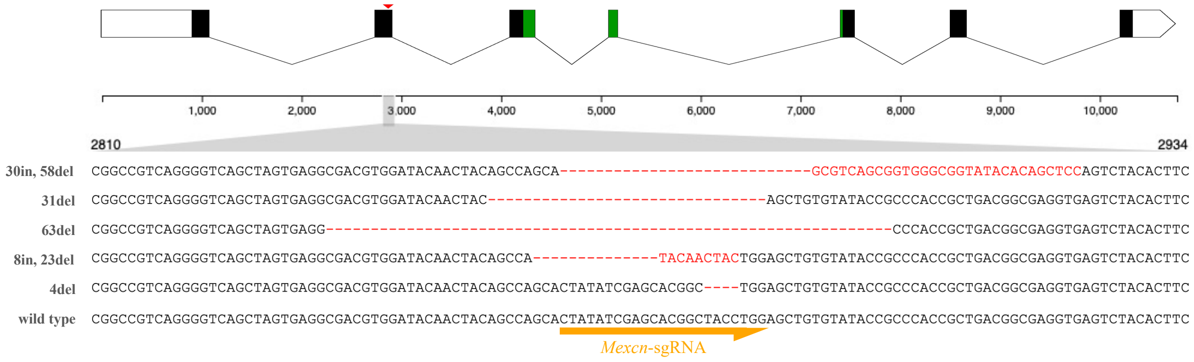

Five different *Mex cn* -/- indels (and no wildtype) were observed in genotyped first instar nymphs (N=17) generated from the mosaic *Mex cn* -/- individual. For comparison the wildtype sequence obtained from the haemolymph of the mosaic animal is shown at the bottom.

Table S1. GenBank accession numbers and reference DOIs for Fig. 1c of the main manuscript

**Species**	***cinnabar***	***white***	**Reference DOI**
A. aegypti	AF325508	-	10.1046/j.1365-2583.2003.00433.x
B. anynana	XM_024096156.1	-	10.1038/s41598-023-36491-9
B. dorsalis	-	XM_011202225.2	10.1111/imb.12592
B. mori	AB063490	AB445460	10.1002/btpr.3054,<br>10.1016/j.ibmb.2008.10.003
C. quinquefasciatus	CPIJ07147	-	10.1371/journal.pone.0224857
D. melanogaster	NM_078927	NM_057439.2	0.1007/BF00483738
F.occidentalis		XM_052276619.1	10.1111/imb.12913
H. armigera	-	KU754476	10.1038/srep40025
H. hebetor		PRJNA644201	10.1002/ps.7851
H. vitripennis	XM_046816083.1		10.1038/s41598-022-09990-4
L. hesperus	MH806848	MH806842	10.1038/s41598-022-08908-4
L. niger	LBMM01007519.1	-	10.1111/imb.12809
N. lugens	KP881329	KU376474	10.1016/j.ibmb.2017.12.003
N. vitripennis	NV14284	-	10.1038/s41598-017-00990-3
P. interpunctella		MN379839.1	10.1016/j.jinsphys.2022.104471
P. xuthus	-	KQ459302	10.1186/s12864-021-07400-z
R. prolixus	-	RPRC012709-RA	10.1093/genetics/iyac064
T. absoluta	OM959367	-	10.3389/fgene.2022.865622

Figure S2. **Snapshots of injection procedure**

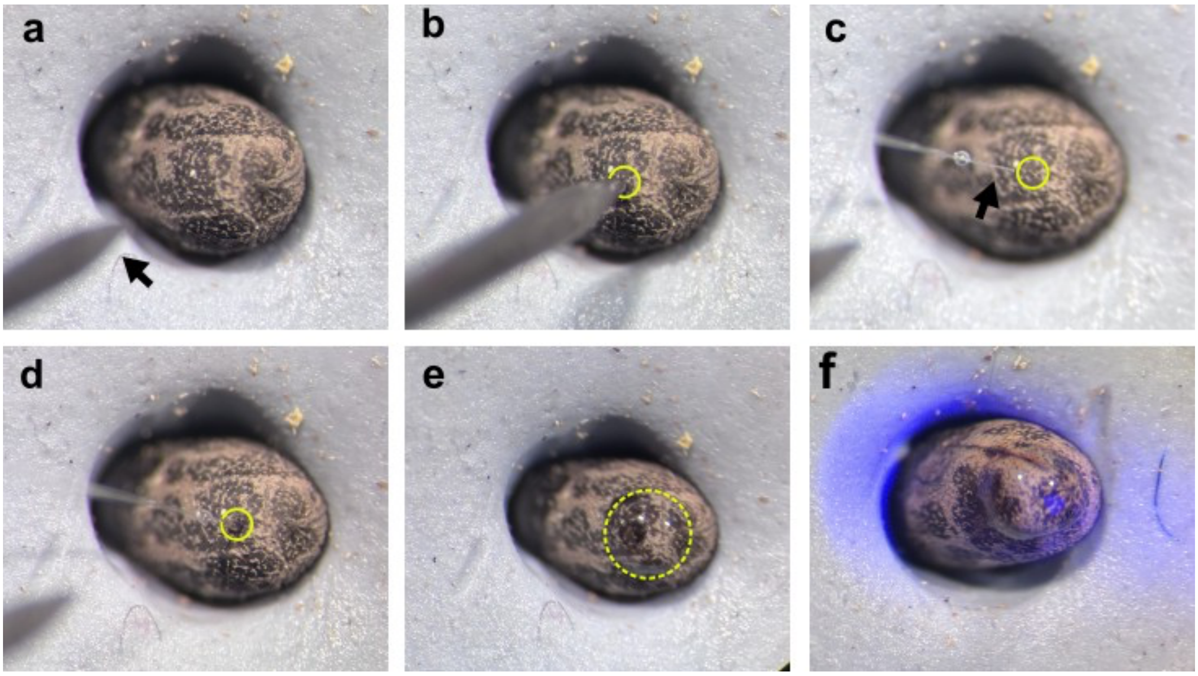

The injection procedure for a single egg is illustrated from (**a**) to (**f**). A single egg is placed in a narrowly fitting depression in BluTack. First, the tip of a tungsten needle (black arrow, (**a**)) is used to pierce the egg's exochorion (**b**). Needle has already penetrated in (**b**), yellow circle surrounds the penetration site. Next, a pulled glass capillary (black arrow in (**c**) capillary is approaching the opening) is inserted into the opening, and the RNP solution is injected into the egg (**d**). Finally, a drop of glue (Loctite AA 3321 LC, inside the dotted yellow circle) is applied to seal the hole (**e**) and hardened using UV light (**f**).

Table S2. List of primers used in this study, sequences are given in 5'-3' direction. Color code for the sgRNA templates is as follows: grey, T7 Polymerase promotor sequence; red, target sequence; blue, Cas9 scaffold oligo.

**sgRNA templates**	
Mex-cn	TTCTAATACGACTCACTATAGCTATATCGAGCACGGCTACCGTTTTAGAGCTAGA
Mex-w	TTCTAATACGACTCACTATAGAAGGCGTCCAGACCCGACGTGTTTTAGAGCTAGA
**Genotyping**	
Mex-cn Fwd	TGACAAACTACTGGGCATCGCCT
Mex-cn Rev	TCGTGCCCTTAGCCGACATGGA
Mex-w Fwd	GCACCATGCTTAGAGCTCCACCG
Mex-w Rev	CAGCACCTTGTCGAAGAGCGCA
**Off-target detection**	
*Mex-cn*OT1 Fwd	TGGTAAACATGAGTCGTGTTGAGGT
*Mex-cn*OT1 Rev	GCTCTCGTGGCCCGATAAACCG
*Mex-cn*OT2 Fwd	CTCCTTTTCTTCTGCCCCGGCG
*Mex-cn*OT2 Rev	TGAGGTGTTTGACGGACGGGGT
*Mex-w*OT1 Fwd	TGCTCCCACAAGCTTACACTTGCA
*Mex-w*OT1 Rev	GCTCGCTAACTGCCTGACTGCC

SI text. Coding sequence for *Mex-cn* and *Mex-w* as predicted from the genome CDS.
Target sequences are highlighted, PAM in blue.

> Mex-cn

ATGAAGGTACACAAAGATACGAAGGTCCTGGGCCGATGTCACGGAATGTGTCAGAGTAAGTACAGAATAGTG
AATTCTAGCCTGTCGGCAACTAATAACGTGAAATTCGCATGTCACTATGCATTAGTACTAACGCTACATGTA
GCCTTGGTTGAGCCAAACAGCTGCAGGACGGGCAGCTCAGATCTGCTACTGCAGCAGGCGGACTTGGTGGTA
GGGGCTGACGGCGCACACTCGGCCGTCAGGGGTCAGCTAGTGAGGCGACGTGGATACAACTACAGCCAGCAC
TATATCGAGCACGGCTACCTGGAGCTGTGTATACCGCCCACCGCTGACGGCGAGTTTGCGATGGAGGCGAAC
CACCTGCACATCTGGCCACGCAACACCTTCATGATGATCGCGCTGCCGAACCAGGACCGCTCCTACACCGTC
ACTCTCTTCATGCCCTTCCAGCAGTTCCAGCAACTGAACGACGCTGACAGTCTGTTGCGCTTCTTCGCCGAG
CACTTCCCAGACTCGCTGCCTCTCATCGGCGAGGAGCAGCTGGTCAACGACTTCTTCGCCAACAAGCCGTCG
CCCCTCGTCAGCGTCAAGTGCAATCCCTACCACTACGGCAGCACGGCAGTCATCATAGGAGACGCAGCTCAC
GCTATGGTGCCGTTTTATGGTCAAGGCATGAATGCGGGGTTCGAGGACTGCCGCCTGCTGAACGAGTTGATG
ACGCGACACCATGACAACATGTCTCTAGTGCTCAGGGAGTTCTCAGAGACGAGAGGCGGGGACGCCGCGGCC
ATCTGCGACCTCGCGCTCTACAACTACGTCGAGATGCGTGACCTGGTGAACAAGAAGTCGTTCATCTTCAGG
AAGAAGCTGGACCAGTTGCTGAACGGGATGTTCCCAAAGTTGTGGGTGCCGCTGTACACCTCGGTCACCTTC
ACCAACACGCCGTACAGCACCTGCCTGCAGCACAAGCGCTTCCAGGACAAAGTGCTGGAAGCTGTGCTGACA
TCTGCGGGCGTGCTGCTGGGGCTGTTTCTGGTCGTCCTGCTGACCCGCTGGTACGTCTGCGAGACTGCCAAG
GACGTCGGTGATCGCCGCATCTACGAGTGA

> Mex-w

ATGTTCTGCGACGAGCCAACGTCGGGTCTGGACGCCTTCATGGCACAGAACGTGGTGTCTGTGTTGAAGAGT
ATGGCAGAGAAGGGCAAGACTGTAGTGTGCACTATACACCAGCCTTCCTCCGAGGTGTTTGCGCTCTTCGAC
AAGGTGCTGCTCATGGCCGAGGGTCGCGTTGCCTTCCTCGGCACCGCCAAGCAGGCCTGCGACTTCTTCCGC
AAGCTAGGAGCGGCTTGTCCTACCAACTACAACCCGGGAGACTTCTTCATCCAGCTGCTAGCCGTCATACCG
TCCCGCGAGGAAGCTTGCAAGGAGACTATAGAGCTGATATGTGACAACTTCCATACCTCCGACATGGGCGTG
CACATACACCAGGTCACAAAGCCCAAACTCTACCAGTCCCTCAAATTACGACGGCGGCAGGAACAGGCGTCA
CAGAATATCGTCTACCGCATACGTCTCGCTCGCGTGGGAGTCGGGAAGAAGCTAAAAGGACGCCGCTGA
